# Ligustrazine Alleviate Acute Lung Injury Through Suppressing Pyroptosis and Apoptosis of Alveolar Macrophages

**DOI:** 10.3389/fphar.2021.680512

**Published:** 2021-05-28

**Authors:** Rundong Jiang, Jiaqi Xu, Yuezhong Zhang, Xuanmeng Zhu, Jiachen Liu, Yurong Tan

**Affiliations:** ^1^ Hunan Engineering Research Center of Skin Health and Disease, Xiangya Hospital, Central South University, Changsha, China; ^2^ Department of Dermatology, Xiangya Hospital, Central South University, Changsha, China; ^3^ Clinical Medicine Eight-Year Program, Xiangya School of Medicine, Central South University, Changsha, China; ^4^ Department of Spine Surgery, Xiangya Hospital, Central South University, Changsha, China; ^5^ Key Laboratory of Organ Injury, Aging and Regenerative Medicine of Hunan Province, Changsha, China; ^6^ Department of Cardiovascular Medicine, The Second Xiangya Hospital, Central South University, Changsha, China; ^7^ Department of Geriatric Medicine, Xiangya Hospital, Central South University, Changsha, China; ^8^ National Clinical Research Center for Geriatric Disorders, Xiangya Hospital, Central South University, Changsha, China; ^9^ Department of Medical Microbiology, China-Africa Research Center of Infectious Diseases, School of Basic Medical Science, Central South University, Changsha, China

**Keywords:** ligustrazine, acute lung injury, NFκB, inflammasome complex, pyroptosis, apoptosis

## Abstract

Ligustrazine (Tetramethylpyrazine, TMP) is an active substance extracted from the Umbelliferae plant Ligusticum chuanxiong. It has been proven to have antioxidant and inflammation effects. The study was designed to explore the efficacy and specific mechanism of TMP for ALI/ARDS treatment. Here, we confirmed that TMP decreased the infiltration of inflammatory cells in alveoli and the secretion of pro-inflammatory factors, which is comparable to glucocorticoids *in vivo*. *In vitro,* TMP inhibited the polarization of M1-type macrophages, and to a certain extent, promoted M2-type repolarization, thus reducing LPS-induced massive transcription and secretion of IL-1β, IL-18, TNF-ɑ and other inflammatory factors. Besides, TMP reduced expression of NLRP3, inhibited the formation of inflammasome complexes, and decreased the cleavage of caspase-1, leading to reduced cell pyroptosis and accompanying inflammation. TMP also inhibited apoptosis through caspase-8/caspase-3 signaling pathways. Our study indicates that TMP improved ALI through inhibiting the TLR4/TRAF6/NFκB/NLRP3/caspase-1 and TLR4/caspase-8/caspase-3 signaling pathways, which reversed macrophages polarization, reduced cell pyroptosis and apoptosis, which provides a theoretical basis of using TMP in treating ALI in the future.

## Introduction

Acute lung injury (ALI) is characterized by the exudation of protein-rich inflammatory fluid, the infiltration of inflammatory cell and the hemorrhage of lung tissues, leading to impaired diffusion, decreased ventilation/blood flow ratio, declined alveolar compliance and other clinical manifestations. Acute respiratory distress syndrome (ARDS) is considered to be converted from ALI when the alveolar oxygen partial pressure/inhaled oxygen fraction (PaO2/F1O2) is less than 300, the fatality rate of which is as high as 30–40% (Wheeler and Bernard, 2007). In the 2012 Berlin conference, ALI was redefined as a mild level of ARDS (Force et al., 2012).

Ligustrazine, also known as tetramethylpyrazine (TMP) with chemical formula of C8H12N2, was reported to inhibit the process of inflammation process. For example, TMP could reverse the activation of NLRP3 inflammasome and caspase-1 and inhibit the expression and secretion of IL-1β in LPS-treated LO2 hepatocytes (Wang et al., 2015); TMP could also reduce the expression of MyD88 to alleviate cell injury and apoptosis caused by LPS in ATDC5 chondrocyte (Zhang et al., 2016). In lung-related studies, TMP could effectively reduce LPS-induced lung injury in mice, including reducing lung wet-to-dry ratio and the expression of pro-inflammatory factors and TLR4 (Ding et al., 2020). Whereas, so far the efficacy and specific mechanism of TMP for ALI/ARDS treatment are still unclear.

It is widely believed that the pathogenesis of ALI is mainly related to infection-induced inflammation. Nucleus Factor Kappa B (NFκB) signaling pathway is involved in the inflammatory response and cell chemotaxis of macrophages, regulating a variety of physiological and pathological processes especially inflammation (Stocks et al., 2018; Wang et al., 2019). NFκB also builds a bridge between pattern recognition receptors (PRRs) such as TLR and inflammasomes. Inflammasome is usually composed of three elements, PRRs such as NLRP3, apoptosis-associated speck-like protein (ASC) containing caspase-recruitment domain (CARD), and caspase-1 precursor (Rathinam and Fitzgerald, 2016). While activation of the inflammasome could convert the precursor caspase-1 into active caspase-1, triggering classic pathway or caspase-1-dependent pathway of pyroptosis (Man and Kanneganti, 2016).

It was reported that apoptosis mediated by caspase-3/7 activation could also be found in Gasdermin D (GSDMD) knockout human monocytes when treated with agonist for completely caspase-1-dependent programmed cell death (Taabazuing et al., 2017). And caspase-3 showed significantly reduction with caspase-1 knockedout in the study of Absent In Melanoma 2 (AIM2) inflammasome (Sagulenko et al., 2018). These studies provided us with the link between pyroptosis and apoptosis in macrophages. However, the pathogenic role of macrophage pyroptosis-apoptosis in ALI is still unclear. Besides whether such pathway could be a target of TMP treatment remains to be studied.

The contributions of this paper can be summarized as follows: TMP might treat ALI/ARDS patients by reducing the pulmonary inflammation in a mechanism of inhibiting TLR4/TRAF/NFκB/NLRP3/caspase-1 and TLR4/caspase-8/caspase-3 signaling pathways in macrophages and reversing the polarization of macrophages from M1 to M2 to reduce cell pyroptosis and inflammatory response activated by NFκB.

## Materials and Methods

### Reagents

Tetramethylpyrazine (TMP) was purchased from Yuanye Bio-Technology .Co, Ltd (Shanghai, China). LPS from *Escherichia coli* 0111:B4 (LPS) and Dexamethasone (Dex) was obtained from Sigma-Aldrich (St.Louis, MO, United States). Primers for qRT-PCR were synthesized in Tsingke Biological Technology .Co, Ltd (Beijing, China). Primer details were listed in Quantitative Real-Time PCR Assay. Antibodies used for Western blot included GAPDH (ab8245), NFκB p65 (ab16502), NFκB P-p65 (ab194726), TRAF6 (ab33915), caspase-1 (ab74279), caspase-1 (ab 1872), caspase-3 (ab90437) from Abcam (Cambridge, United Kingdom), and β-actin (3700s), ASC (67824T), NLRP3 (1510s), caspase-8 (4927T) from Cell Signal Technology (Danvers, MA, United States), TLR4 (66,350-1-lg) from proteintech (Rosemont, IL, United States). Anti-rabbit (7074s) and anti-mouse (7076s) secondary antibodies conjugated with HRP were from Cell Signal Technology (Danvers, MA, United States). Antibodies used for Immunofluorescence contained iNOS (18985-1-AP), CD206 (18704-1-AP) and CD68 (66231-2-Ig) were purchased from Proteintech Group (Rosemont, IL, United States). Secondary antibodies conjugated with FITC (a0568), and DAPI staining solution (c1006) were from Beyotime (Shanghai, China).

### Cell Culture

Mouse macrophage cell lines, RAW 264.7 and Ana-1 cells were purchased from American type culture collection (Manassas, VA, United States) and cultured in 90% high glucose Dulbecco’s Modified Eagle Medium (DMEM) and RPMI medium 1640 separately (Sigma-Aldrich Corp, St.Louis, MO, United States) with 10% fetal bovine serum (FBS) (Gibco, Carlsbad, CA, UnitedStates) in humidified atmosphere containing 5% CO_2_ and 95% O_2_ at 37°C.

### Cell Proliferation Assay

The RAW264.7 cells were plated into 96-well plates at a density of 1 × 10^4^ cells each well for 24 h and then incubated for another 24 h in the presence of various concentrations of LPS (0.125, 0.25, 0.5, 1, 2 μg/ml) or presence of TMP separately (2.5, 5, 10 μg/ml). The cell viabilities were measured using the Cell Counting Kit-8 (Beyotime, Shanghai, China) method according to the manufacturer’s instruction.

### Morphological Changes of RAW264.7

RAW264.7 cells were seeded at 2 × 10^5^ per well in the 6-well plates during logarithmic growth. Five groups including control group, LPS group, LPS + TMP (5 μg/ml) group, LPS + TMP (10 μg/ml) group and LPS + Dex (5 μM) group were set up respectively. Morphological changes of RAW264.7 cells were observed under inverted light microscopy (Carl Zeiss AG, Oberkochen, Germany) after incubating for 24 h.

### Cell Apoptosis Assay

For cell apoptosis evaluation, RAW264.7 cells were planted into 6-well plates with 2 × 10^5^ cells each well for 24 h and then treated as Control, LPS (1 μg/ml), LPS (1 μg/ml) + TMP (5 or 10 μg/ml) and LPS (1 μg/ml) + Dex (5 μM) respectively. RAW264.7 cells were blown off with PBS from 6-well plates and washed in PBS two times. After incubation with PI and Annexin V with eBioscience™ Annexin V-FITC Apoptosis Detection Kit (Invitrogen, Carlsbad, CA, United States) according to instructions, the cells were analyzed using flow cytometry.

### LPS-Induced ALI Model in Mice

Male C57BL/6J mice 6–8 weeks old, weighing 18–22 g (*n* = 24) were purchased from Hunan Tianqin Biological Technology Co., Ltd. Pentobarbital sodium 35 mg/kg was intraperitoneally injected in order to make mice anesthetized. Mice were randomly divided into the following four groups (*n* = 6): Control group (0.2 ml of 0.9% saline intraperitoneally injected), LPS group (30 mg/kg of LPS intraperitoneally injected), Dex group (5 mg/kg of Dex 1 h before LPS intraperitoneally injected) and TMP group (50 mg/kg of TMP 1 h before LPS intraperitoneally injected). After the treatments for 6 h, blood samples were taken from the eyeballs. BALF and lung tissue were collected and various indexes were measured.

### Survival Rate Analysis

To observe the protective effect of TMP on ALI, another 40 of C57BL/6J mice were randomly divided into four groups with 10 mice in each group. After treatment, the mental state and survival of mice in each group were recorded after observing once every 8 h. The experiment ended at 72 h. The survival rate was calculated and the survival curve was drawn. The Kaplan-Meier curve was used to calculate the survival rate, and the log-rank test was used to compare the significant differences among different groups. *p* < 0.05 was defined as statistically significant.

### Histopathological Evaluation

Fresh tissues from the inferior lobe of left lung were collected from the mice. The tissues were fixed with 4% paraformaldehyde for 24 h. After alcohol gradient dehydration and dewaxing, paraffin-embedded sections with a thickness of 4 μm were prepared. The pathological changes of lung tissues were observed under light microscope after HE staining.

### Measurement of Myeloperoxidase (MPO) in Lung Tissues

About 100 mg of lung tissues were homogenized in 1 ml of pre-cooled PBS (pH = 7.4), and the supernatant was collected after centrifuging at 12,000 g at 4°C for 10 min. The contents of MPO in the lung tissues were detected according to the instructions of MPO ELISA kit (Nanjing Chengchang Company, Nanjing, China).

### Cell Counting and Total Protein Concentrations in BALF

The mice were fixed, and the skin and muscles in front of the neck were cut in order to expose the neck trachea. Endotracheal intubation and BALF were collected with 0.5 ml of saline for 3 times and BALF were gathered together. After centrifuging BALF at 4°C for 10 min at 3,500 rpm, the precipitation was resuspended and the red blood cells were removed with 1 ml of red blood cell lysate by centrifuging at 4°C for two times at 3,500 rpm for 10 min. The precipitation was resuspended with 100 μl of normal saline. Wright-Giemsa staining was used for cell classification and counting. BCA protein concentration assay kit (Beyotime, Shanghai, China) was used to determine the protein contents of the retained supernatant of BALF.

### Quantitative Real-Time PCR Assay

Macrophage cell lines, RAW264.7 and Ana-1 cells (2 × 10^5^ cells in a 6-well plate) were cultured with LPS (1 μg/ml), LPS (1 μg/ml) + TMP (10 μg/ml) and LPS (1 μg/ml) + Dex (5 μM) for 24 h at 37°C in a humidified air atmosphere with 5% CO_2_. Total RNA was extracted with TRIzol Reagent (Beyotime, Shanghai, China) and 1 μg of RNA was used to synthesize cDNA through Hifair® III 1st Strand cDNA Synthesis Kit (YEASEN, Shanghai, China) after quantitating on a spectrophotometer. In addition, the mouse lung tissues were collected and treated in the same way. qRT-PCR was performed using Hieff UNICON® qPCR SYBR Green Master Mix (YEASEN, Shanghai, China) in a RT-PCR machine (ViiA™ seven system, Thermo Fisher Scientific, Waltham, MA, United States). The relative levels of mRNA expression were calculated as ΔCt = Ct (target) -Ct (reference). 2^−△△Ct^ reflected the target gene expression levels relative to the control sample. The forward (F) and reverse (R) primers for the tested genes were provided in Table 1.

**TABLE 1:** Primers used for the qRT-PCR study.

Gene	Forward primer (5'→3′)	Reverse primer (5'→3′)
TNF-α	CAG​GCG​GTG​CCT​ATG​TCT​C	CGA​TCA​CCC​CGA​AGT​TCA​GTA​G
IL-1β	GCA​ACT​GTT​CCT​GAA​CTC​AAC​T	ATC​TTT​TGG​GGT​CCG​TCA​ACT
IL-18	GAC​TCT​TGC​GTC​AAC​TTC​AAG​G	CAG​GCT​GTC​TTT​TGT​CAA​CGA
IL-10	GCT​GGA​CAA​CAT​ACT​GCT​AAC​C	ATT​TCC​GAT​AAG​GCT​TGG​CAA
Caspase-1	ACA​AGG​CAC​GGG​ACC​TAT​G	TCC​CAG​TCA​GTC​CTG​GAA​ATG
NLRP3	ATC​AAC​AGG​CGA​GAC​CTC​TG	GTC​CTC​CTG​GCA​TAC​CAT​AGA
iNOS	GGA​GTG​ACG​GCA​AAC​ATG​ACT	TCG​ATG​CAC​AAC​TGG​GTG​AAC
IL-6	TAG​TCC​TTC​CTA​CCC​CAA​TTT​CC	TTG​GTC​CTT​AGC​CAC​TCC​TTC
CD206	CTC​TGT​TCA​GCT​ATT​GGA​CGC	CGG​AAT​TTC​TGG​GAT​TCA​GCT​TC
TLR4	ACA​CCT​GCC​TCT​TCC​CTC​CC	CTC​CAG​TCG​GTC​AGC​AAA​CG
GAPDH	AGG​TCG​GTG​TGA​ACG​GAT​TTG	GGG​GTC​GTT​GAT​GGC​AAC​A

### Enzyme-Linked Immunosorbent Assay (ELISA)

The supernatant of cell cultures were collected. *In vivo* experiment, the blood samples extracted from eyeballs were placed for 0.5 h at 37°C and centrifuged at 3,500 rpm at 4°C for 10 min. The IL-18, IL-1β, IL-10, TNF-α were measured using ELISA Kits (Abcam, Cambridge, United Kingdom). according to the manufacturer’s instructions at OD450 value immediately with Bio-Rad model 550 microplate reader (Bio-Rad, Hecules, CA, United States).

### Western Blot Assay

Macrophage cell lines, RAW264.7 and Ana-1 cells (3 × 10^5^ cells in a 6-well plate) were co-cultured with LPS (1 μg/ml) and TMP (10 μg/ml) or Dex (5 μM) for 24 h. *In vivo* experiments, the lung tissues were dissolved into lysates in RIPA Lysis Buffer (Beyotime, Shanghai, China) with a Phenylmethanesulfonyl fluoride and phosphatase inhibitor (Beyotime, Shanghai, China). Equal amounts of protein (50 μg/lane) were separated electrophoretically through SDS-PAGE (10%, 12%) and transferred onto a PVDF membrane (Millipore Corp, Billerica, MA, United States). The PVDF membranes were then blocked for 2 h at room temperature with 5% non-fat milk in TBST. The membranes were incubated with appropriate primary antibodies (1:1000) in QuickBlock™ Primary Antibody Dilution Buffer (1:1,000, Beyotime, Shanghai, China) for overnight at 4°C and horseradish peroxidase-conjugated secondary antibodies 1:1,000 with 1% non-fat milk for 2 h at room temperature. Images were digitally acquired with chemiluminescence image analysis system (Tanon, Shanghai, China). The expression levels of target proteins were normalized with β-actin or GAPDH as an internal control.

### Flow Cytometry Analysis

The expression levels of F4/80, CD80, CD86 and CD206 in RAW264.7 cells were analyzed using flow cytometry. After treatment, cells were harvested and washed in PBS and then stained with F4/80(APC), CD80(APC), CD86(BV650), and CD206(BV421), (1:2,00, Biolegend, San Diego, CA, United States) for 30 min at 4°C avoiding from light. After incubation, cells were washed three times with PBS and then analyzed by a BD LSRFortessa X-20 flow cytometer (BD Biosciences, Franklin Lakes, NJ, United States). M1 macrophage cells ratio was calculated as F4/80^+^CD80^+^CD86^+^ and M2 macrophage cells ratio was calculated as F4/80^+^CD206^+^.

### Immunofluorescence Assay

For the immunofluorescence staining of iNOS and CD206, the cells or lung tissues were fixed with 4% paraformaldehyde for 1 h and then incubated using primary antibodies including anti-iNOS, anti-CD206 and anti-CD68 for labeling the macrophages in lung tissues (BD Biosciences, Franklin Lakes, NJ, United Statse) in a humidified chamber at 4°C overnight. After washing twice with PBS, the cells or tissues were incubated with a fluorescein-labeled secondary antibody for 2 h avoiding light (Beyotime, Shanghai, China). Eventually, cell nuclei were stained with DAPI for 0.5 h (Beyotime, Shanghai, China). The immunofluorescence images were captured using a fluorescence microscope (Leica Microsystems, Weztlar, Germany).

### Protein Extraction

RAW264 cell was co-cultured with LPS (1 μg/ml) and TMP (10 μg/ml) for 24 h. The cell samples including Control, LPS (1 μg/ml) and LPS (1 μg/ml) + TMP (10 μg/ml) groups were added with 1,000 μl of RIPA lysis buffer and ultrasonicated in the ice bath for 5 min until they were fully dissolved (Kangchen Bio-tech, Shanghai, China). Then, the protein solutions were centrifuged at 14,000 g for 15 min at 4°C and performed BCA quantification for the supernatant. Then 4–6 times of pre-cooled acetone was added to the samples and placed on ice for 30 min. After that, the proteins were precipitated at 4°C, 10,000 g for 10 min. After the supernatants were removed, the precipitations were resolved, reduced, and alkylated, and removed sodium deoxycholate (SDC). Next, after 500 μl of ACN (Acetonitrile) was used to equilibrate the C18 desalting column, 1 ml of Buffer A (0.1% FA, H_2_O, 2% ACN) was added to wash away the residual ACN and activate the desalting column. The samples were then added to the desalting column, centrifuged at low speed and the effluent A was collected after repeating once. After washing the column with 1 ml of Buffer A once to remove residual salt, 400 μl of Buffer B (0.1% FA, 70% ACN) was used to elute the peptides. The effluent B was collected and then vacuum dried. The peptides were resolved in buffer A to 1 μg/μl and freezen at −80°C until use.

### Phosphopeptide Enrichment and Phosphopeptide Identification

After preparing TiO2 bead(GL Sciences) and protein mixtures (W:W = 6:1), they were resuspended in 200 μl loading buffer (pho-L): 80% ACN (Acetonitrile)/6%TFA (Trifluoroacetic acid)/saturated PA (Phthalic acid). The mixture were then transferred to a C8 stage tip and centrifuged at low speed until no liquid remains. Then the beads were washed 5 times with washing buffer (pho-W) (60%ACN/1%TFA) with 200 μl each for 4 times and 100 μl for the last time. After 50 μl of Elution buffer 1 (pho-E1:10% NH4OH) and Elution buffer 2 (pho-E2:5% NH4OH/50% ACN) were added to elute the phosphorylated peptide twice and once, respectively, the phosphorylated peptides eluted three times were combined, dried in vacuum at room temperature, and dissolved in 0.1% FA for subsequent LC/MS analysis.

Half of the phosphorylated peptides from each sample were separated by the nano-UPLC liquid system EASY-nLC1200, and detected by the online mass spectrometer Q-Exactive (Thermo Scientific, MA, United States). We used a 100 μm ID × 15 cm reversed-phase chromatography column (Reprosil-Pur 120 C18-AQ, 1.9 μm, Dr. Math) for the analysis. The mobile phase A liquid was 0.1% formic acid acetonitrile aqueous solution (ACN was 2%) and B liquid was 0.1% formic acid acetonitrile aqueous solution (ACN was 80%). The column was equilibrated with 100% A liquid. The samples were directly loaded onto the chromatographic column by the autosampler and then separated by the chromatographic column. The flow rates were 300 nL/min and the gradient durations were 240 min. Mobile phase B was 8–35% for 184 min, 35–45% for 40 min, 45–100% for 4 min, 100% for 4 min, 100–2% for 4 min and 2% for 4 min. The enzymatic hydrolysis products were separated by nano-UPLC and then connected to Q-Exactive mass spectrometer (Thermo Finnigan) for online mass spectrometry analysis. Analysis time was 240 min/sample with positive ion detection mode and precursor ion scan range (350–1,600 m/z). The DDA acquisition (20 fragment maps (MS2 scan, HCD) were collected after each full scan (full scan). MS1 had a resolution of 70,000at m/z 200 and MS2 had a resolution of 35,000at m/z 200; MS1 AGC was 3E + 6, MS2 AGC was 1E + 5, and the maximum ion injection time (Max IT) was 50 ms for MS1 and 45 ms for MS2. The standardized collision energy (NCE) was 32%, the isolation window was 2 m/z, and the dynamic rejection time was 40 s. Statistical analysis of the standardized quantitative results was performed to obtain the corresponding differential expression sites. This experiment contained biological repetitions. The expression fold difference (ratio A/B > 1.5, *p*.value <= 1) was defined as significant difference, and subsequent GO, KEGG pathway, protein interaction analysis were performed.

### Statistical Analysis

All the experiments were performed for at least three times. The results were expressed as the mean ± standard error of the mean (mean ± S.E.M). *T-test* was used for comparison between two groups, two-way ANOVA was used for comparison among multiple groups, and the least significant difference (LSD) was used for post hoc test. *p* values <0.05 were considered significant. All statistical analyses were carried out using the GraphPad Prism 7 (GraphPad Software, La Jolla, CA, United States).

## Result

### TMP Played a Protective Role in LPS-Induced Cell Damage

**FIGURE 1:**
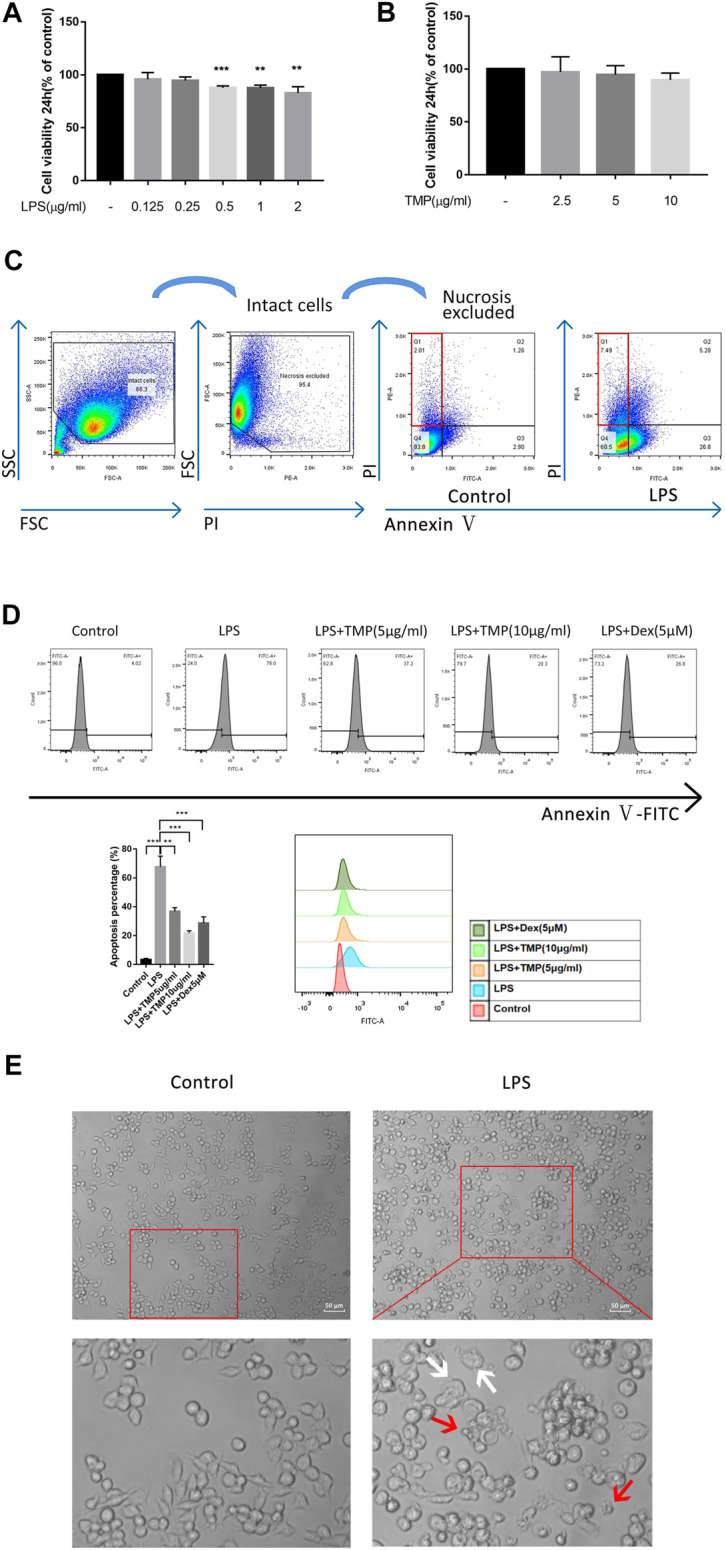
TMP played a protective role in LPS-induced cell damage (*n* = 3). **(A)** RAW264.7 cells were treated with different concentrations of LPS for 24 h and then detected by CCK-8 assay. **(B)** CCK-8 assay was used to detect the cytotoxicity of TMP to RAW264.7 cells. **(C)** The PI- positive rates of RAW264.7 cells were detected using flow cytometry after 24 h stimulation with LPS (1 μg/ml). **(D)** The apoptosis rates of RAW264.7 cells were detected using flow cytometry when LPS (1 μg/ml) were co-treated with TMP (10 μg/ml) or Dex (5 μM) for 24 h. **(E)** After 24 h of treatment with LPS (1 μg/ml), the cells were observed under an inverted optical microscope. The red arrow showed the formation of apoptosis bodies, and the white arrow showed the cell swelling or the rupture of the cell membrane. Averages ±SD were shown. *p < 0.05, **p < 0.01, ***p < 0.001.

At present, many studies have shown that LPS can promote cell apoptosis and pyroptosis of RAW264.7 cells (Ju et al., 2018). Using CCK-8 experiments, we confirmed that LPS in excess of 0.5 μg/ml could produce significant toxic effects on cells and LPS (1 μg/ml) was used as the treatment concentration for subsequent link to figure experiments (Figure 1A). In order to further understand the appropriate treatment concentration of TMP, we also carried out the CCK-8 experiment and found that the concentration of TMP below 10 μg/ml had no significant toxic effect on RAW264.7 cells (Figure 1B), hence, TMP (10 μg/ml) was used as the subsequent experimental treatment concentration. The results of flow cytometry showed that LPS also significantly upregulated PI + Annexin-cell ratio (Figure 1C). In addition, the anti-apoptotic effect of TMP (10 μg/ml) was even better than that of Dex (Figure 1D). The cell morphology of the RAW264.7 cells in the control group had an oval appearance.However, after LPS (1 μg/ml) treatment, polygonal, swelling or vesicle - like changes occurred in cells, and even cell membrane rupture and the formation of apoptotic bodies (Figure 1E), which were considered to be a morphological feature of pyroptosis (Yokoyama et al., 2018). According to the study preformed by Hang Xi (Xi et al., 2016), after excluded necrosis cells based on small size (FSC) and minimal DNA florescence, the PI + Annexin-cell could be considered as pyroptosis. Thus when combining previous results that LPS could induce PI + Annexin-cell with morphological feature, we tend to conclude that this phenomenon might be explained as the pyroptosis of macrophage.

### TMP Regulated Expression of Inflammatory Cytokines of Macrophage When Stimulated by LPS

**FIGURE 2:**
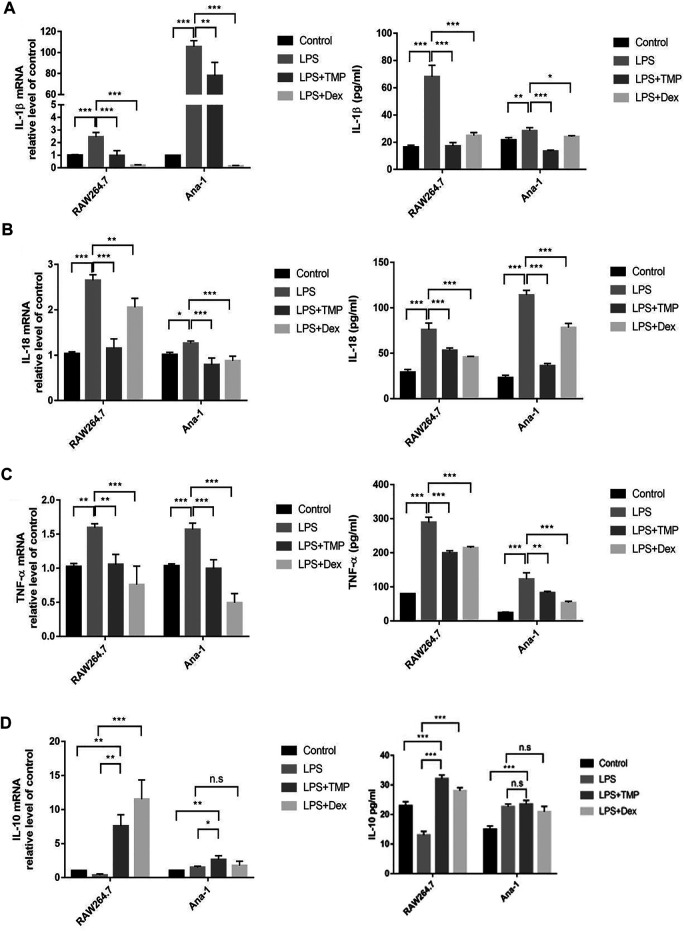
TMP regulated expression of inflammatory cytokines of macrophage when stimulated by LPS (*n* = 3). After RAW264.7 and Ana-1 cells were co-cultured with LPS (1 μg/ml) and TMP (10 μg/ml) or Dex (5 μM) for 24 h, IL-1β **(A)**, IL-18 **(B)**, TNF-ɑ **(C)** and IL-10 **(D)** mRNA expression level were detected by qRT-PCR. ELISA method was used to detect IL-1β **(A)**, IL-18 **(B)**, TNF-ɑ **(C)** and IL-10 **(D)** secretion. Averages ±SD were shown. *p < 0.05, **p < 0.01, ***p < 0.001, n.s = no significance.

To study the anti-inflammatory effects of TMP on LPS-treated mouse macrophage RAW264.7 and Ana-1 cells, we co-treated the cells with LPS or TMP for 24 h, while using the traditional anti-inflammatory drug dexamethasone (Dex) as a positive control. The results demonstrated that TMP played anti-inflammatory roles in the two kinds of macrophages and suppressed the transcriptions and releases of inflammatory factors. Interestingly, TMP was even more effective than Dex in inhibiting the expression of IL-1β and IL-18 (Figures 2A–C). It was worth noting that under the stimulation of LPS, a large amount of IL-1β was transcribed in the Ana-1 cells, but the release of IL-1β in the Ana-1 cells was relatively low. Existing studies had shown that the releases of IL-1β, IL-18 and the occurrence of pyroptosis were depended on GSDMD and activated caspase-1. Only when IL-1β or IL-18 were cleaved by caspase-1 and activated, they could be released outside the cells (He et al., 2015). This indicated that LPS might not induce sufficient activation of caspase-1 in the Ana-1 cells to cleave IL-1β precursors and release IL-1β, indicating that Ana-1 cells might not be the ideal cell model for pyroptosis. In contrast, LPS stimulation could cause a large amount releases of IL-1β and IL-18 from RAW264.7 cells, which supported our idea that LPS could induce pyroptosis of RAW264.7 cells. Moreover, we further examined the changes in the transcription level and the release of IL-10 and found TMP significantly increased the transcription and release of IL-10 from macrophages when compared with the LPS-treated group and the Control group (Figure 2D).

### TMP Inhibited M1-Type and Promoted M2-Type Polarization to Inhibit Inflammatory Responses

**FIGURE 3:**
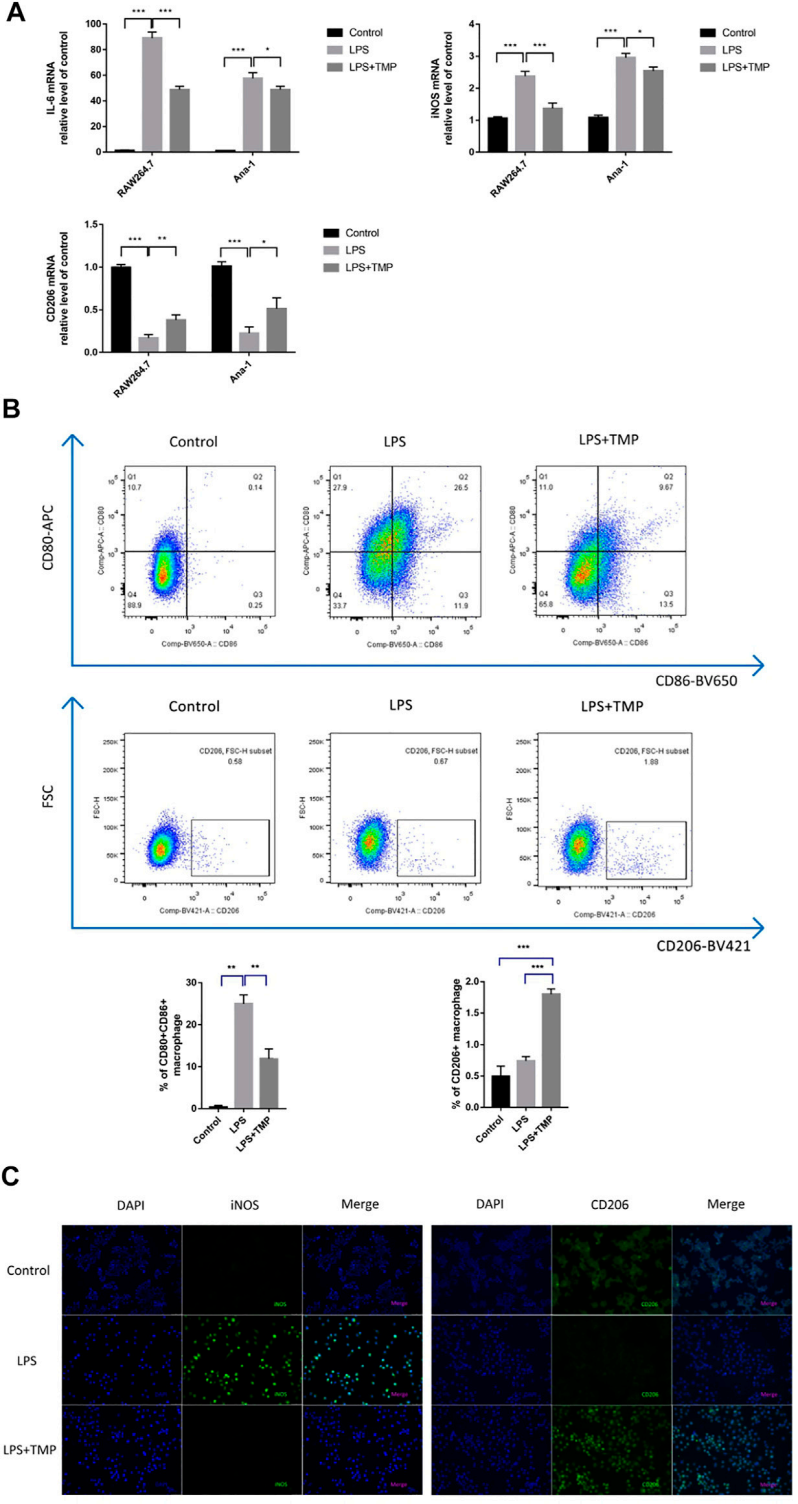
TMP inhibited M1-and promoted M2-type polarization to inhibit inflammatory responses (*n* = 3). RAW264.7 and Ana-1 cells were exposed to TMP (10 μg/ml) or Dex (5 μM) and LPS (1 μg/ml) for 24 h. **(A)** the qRT-PCR experiment was performed to determine M1-type biomarker (IL-6, iNOS) and M2-type biomarker (CD206) expressed in RAW264.7 and Ana-1 cell. **(B)** After RAW264.7 cells were stained with CD80-APC/CD86-BV650 (M1) and CD206-BV421 (M2), the expression levels of macrophage subsets were evaluated using flow cytometry. The figure below showed the quantitative expression of the phenotype of M1/M2 macrophages. **(C)** Immunofluorescence staining was used to detect M1-type macrophage biomarker iNOS and M2-type macrophage biomarker CD206. Blue was DAPI and green was iNOS or CD206. Averages ±SD were shown. *p < 0.05, **p < 0.01, ***p < 0.001.

For further exploring the anti-inflammatory mechanism of TMP, we explored whether the anti-inflammatory effects of TMP were accompanied by inhibiting the expression of M1-type macrophages while increasing the expression of M2-type macrophages. The results of qRT-PCR experiments proved that LPS increased the expression levels, while TMP reduced the expression levels of M1-type biomarkers, including IL-6 and iNOS. More importantly, TMP slightly increased the mRNA level of the CD206 (Figure 3A), which was the typical biomarker of M2-type macrophages. Consistent with the results of the qRT-PCR study, the results of flow cytometry showed that LPS could significantly induce macrophages to polarize to M1 type, and CD80^+^CD86^+^(M1) cells were increased. After TMP treatment, compared with LPS group, the proportion of M1-type (CD80^+^CD86^+^) macrophages decreased from 26.5 to 9.67%, while the proportion of M2-type (CD206^+^) macrophages increased from 0.67 to 1.88% (Figure 3B). On the other hand, the results of immunofluorescence staining showed that LPS could significantly induce the high expression of iNOS (M1) while inhibiting the expression of CD206 (M2) in RAW264.7 cells. After TMP treatment, the expression of iNOS (M1) was significantly reduced, while the expression of CD206 (M2) in RAW264.7 cells was significantly enhanced compared to the LPS group (Figure 3C).

### TMP Had a Protective Role on LPS-Induced ALI in Mice

**FIGURE 4:**
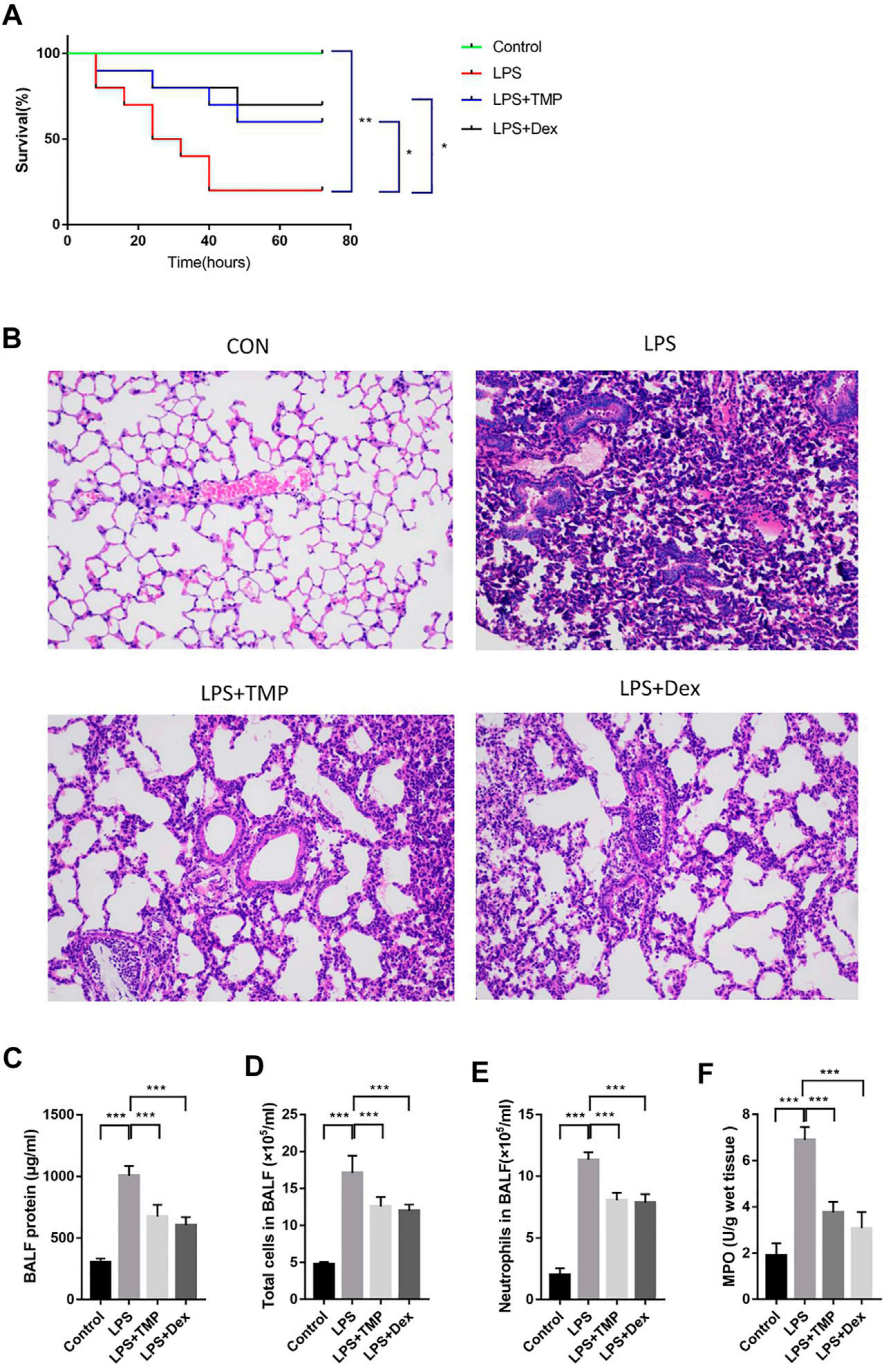
TMP had a protective role in LPS-induced ALI in mice (n ≥ 5). C57BL/6J male mice (18–22 g) aged 6–8 weeks were randomly divided into control group,LPS (30 mg/kg) group, TMP (50 mg/kg) group and Dex (5 mg/kg) group. **(A)** The survival time of mice was monitored for 72 h, and each point on the line represents the cumulative mortality. **(B)** After LPS administration for 6 h, lung tissue samples were collected and tissue sections were made and stained with Hematoxylin and Eosin. **(C-E)** BALF specimens were collected at 6 h after treatments. After Giemsa staining, neutrophils were counted. **(F)** Detection of the inhibitory effect of TMP on myeloperoxidase (MPO) activity. Averages ±SD were shown. *p < 0.05, **p < 0.01, ***p < 0.001.

With the propose of verifying the anti-inflammatory effects of TMP in ALI animal models, We examined the survival rate of the mice treated with different drugs and found that the mice in the control group all survived when the experiment ended at 72 h, while the survival rate of the mice in LPS group was only 20%. The survival rate of mice pretreated with TMP increased to 60% (Figure 4A). To further understand the indicators of inflammatory damages, we collected lung tissue samples at 6 h after LPS treatment and stained the sections with Hematoxylin and Eosin to determine the extent of lung damage *in vivo*. After the administration of LPS alone, it was found that the lung tissue was significantly damaged with pulmonary interstitial edema and hemorrhage, alveolar wall thickness, and inflammatory cell infiltration (Figure 4B). TMP could effectively alleviate these inflammatory reactions, especially reducing inflammatory cell infiltration. Next, we obtained the mouse alveolar lavage fluid (BALF). The protein concentration, total cell number, and neutrophil number in BALF of the LPS group increased significantly, which were inhibited by TMP or Dex. Moreover, from the quantitative data, it was found that TMP had the anti-inflammatory function as effective as Dex (Figures 4C–E). Furthermore, MPO is often used as one of the important indicators of inflammation (Hickey, 2011). In contrast to the Control group, the MPO activities of the LPS group were much higher, and this increment was significantly reduced after TMP pretreatment (Figure 4F).

### TMP Regulated Polarization of Alveolar Macrophages to Inhibit Releases of Pro-Inflammatory Cytokines

**FIGURE 5:**
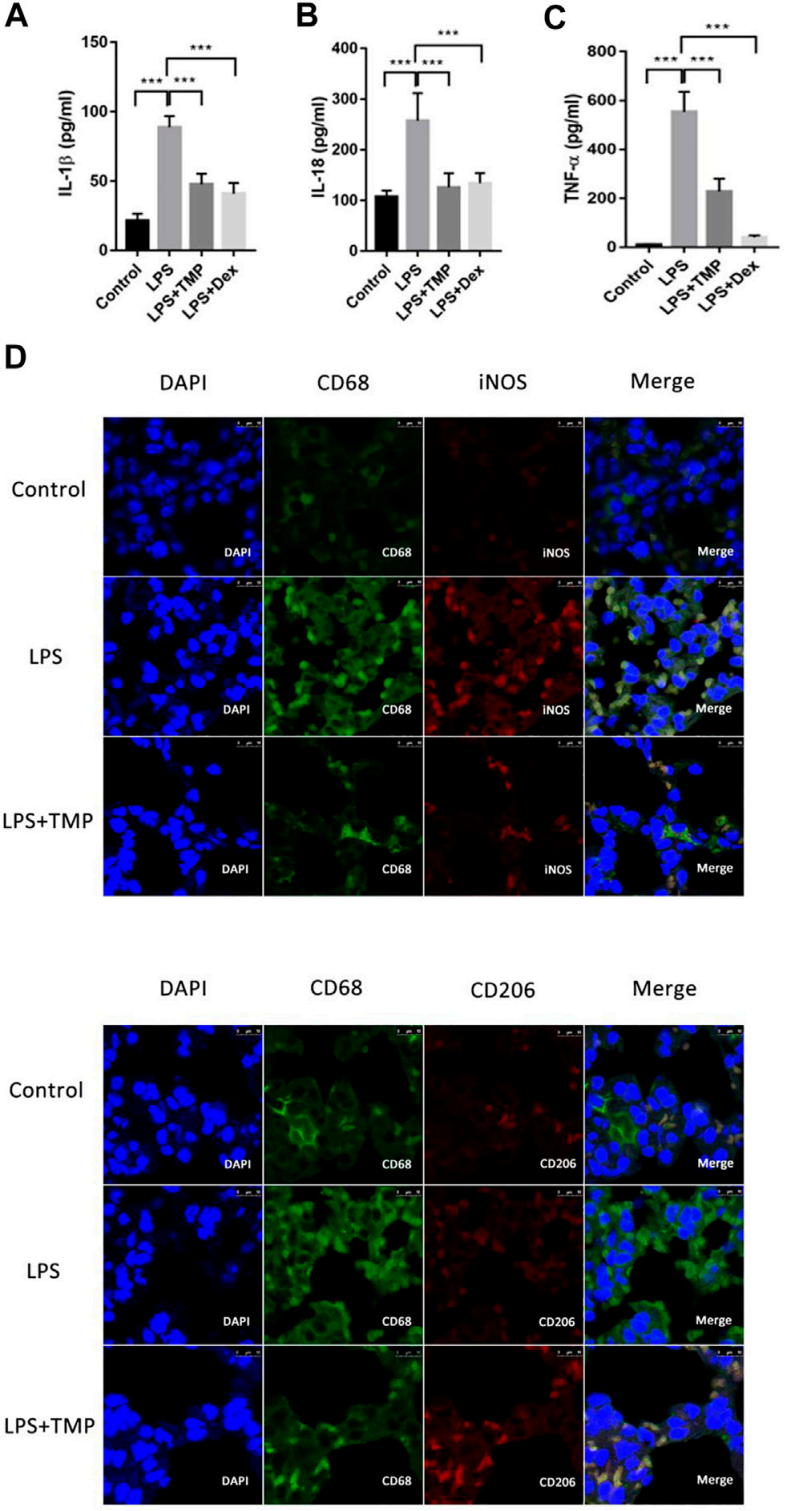
TMP inhibited the releases of pro-inflammatory cytokines from lung tissue through regulating the polarization of alveolar macrophages (n ≥ 4). Mice were randomly divided into control group, LPS group, LPS + Dex group and LPS + TMP group. After 6 h of treatments, venous blood samples were collected from the mice and the serum inflammatory factors including IL-1β **(A)**, IL-18 **(B)**, and TNF-ɑ **(C)** were detected using ELISA method. **(D)** After the mice were sacrificed, the lung tissues of the mice were taken to make paraffin sections, and immunofluorescence was used to label the polarization of macrophages. DAPI was used for nuclear staining, the green fluorescent labeled CD68, the red fluorescent-labeled iNOS or CD206, and merge figures were the overlay of CD68 and iNOS or CD206. Averages ±SD were shown. *p < 0.05, **p < 0.01, ***p < 0.001.

To evaluate the therapeutic effects of TMP on the releases of pro-inflammatory cytokines *in vivo*, the levels of IL-18, TNF-α and IL-1β in the serum were detected using ELISA kits. LPS increased the levels of IL-18, IL-1β, and TNF-α in the serum (Figures 5A–C), while TMP effectively reduced the levels of IL-18 and IL-1β in the serum, and its anti-inflammatory effects were similar to those of Dex. Furthermore, TMP effectively restrained the release of TNF-α. To better evaluate the polarization of macrophages in lung tissue *in vivo*, nitric oxide synthase (iNOS) and mannose receptor (CD206) antibodies were used as the biomarkers of alveolar macrophages. The results showed that LPS caused substantial macrophages (CD68) to infiltrate into lung tissue and increased the expression of iNOS (M1) in macrophages, while TMP selectively decreased the infiltration of macrophage as well as the expression of iNOS (M1) . Moreover, when compared with control group,TMP somehow enhanced the expression of CD206 on the surface of macrophages. Although the CD206 staining seemed positive in the LPS and the control group, but the fluorescence intensity of CD206 in TMP group was much stronger in general (Figure 5D).

### Phosphorylation Protein Profiling Analysis Revealed Inflammation Signaling Pathways Regulated by TMP

**FIGURE 6:**
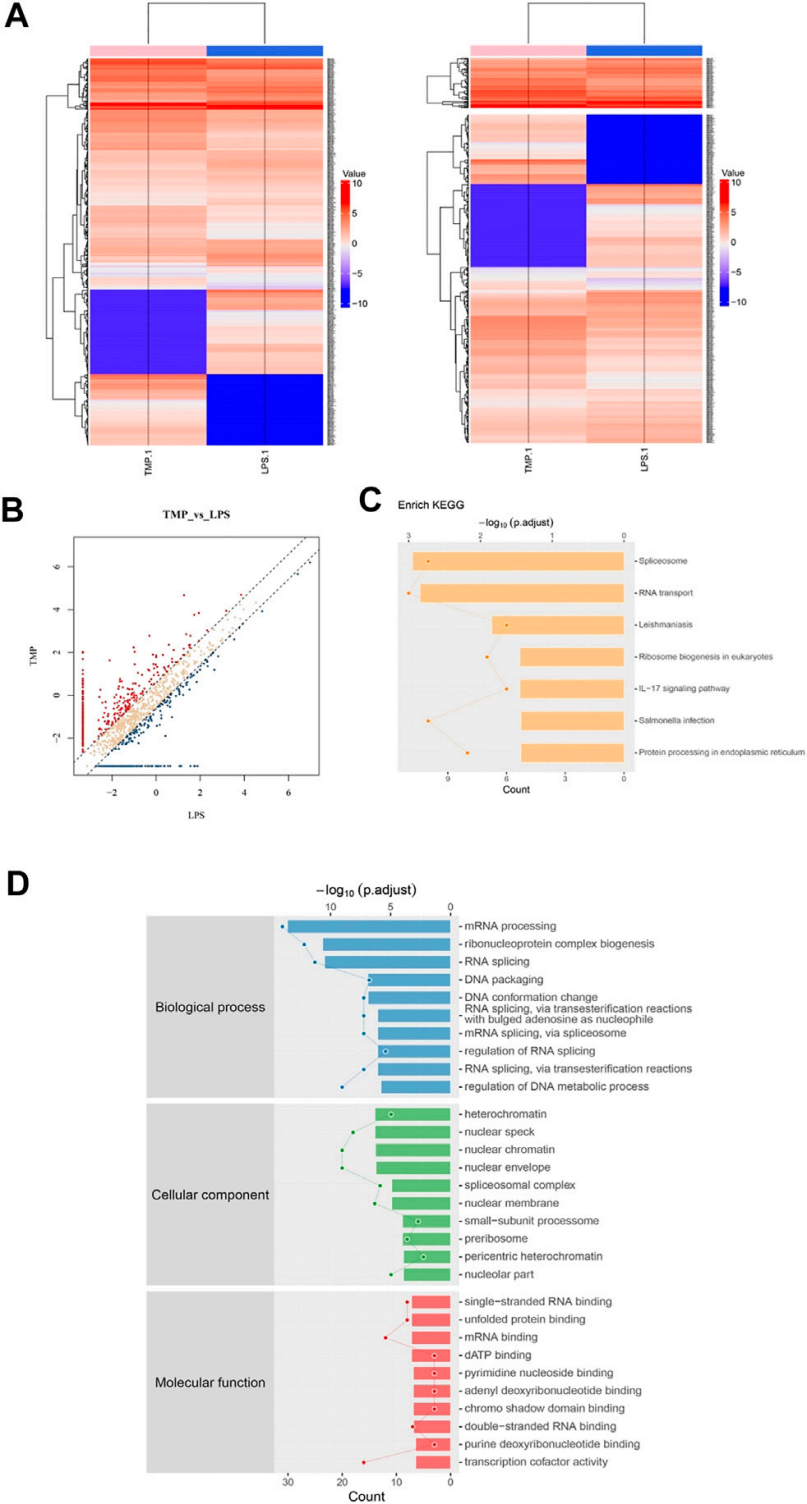
Phosphorylation protein profiling analysis revealed inflammation signaling pathways regulated by TMP. Three groups of Control, LPS (1 μg/ml), LPS (1 μg/ml) + TMP (10 μg/ml) after 24 h of treatments were collected for phosphorylation protein profiling analysis. **(A)** Heat map of phosphorylated proteins with differentially expressed between LPS and TMP, red indicated high expression, blue indicated low expression, and colorless indicated no significant difference. A total of 370 differentially expressed proteins were detected. **(B)** The scatter diagram displayed differentially expressed proteins, and the points distributed outside the two longitudinal boundary lines and above the horizontal boundary represented significantly different proteins. **(C)** KEGG pathway analysis showed the signal pathway enriched by down-regulated proteins. **(D)** GO analysis of target genes in biological processes showed that down-regulated proteins were more abundant in mRNA processing, ribonucleoprotein complex biogenesis, and RNA splicing.

For the sake of fully understanding the inflammatory signal pathways involved in the anti-inflammatory mechanism of TMP, we focused on the phosphorylated proteins that were down-regulated in TMP group compared with those in the LPS group using phosphorylation protein profiling analysis. After understanding the post-translational modification (phosphorylation) of proteins (Figures 6A,B), KEGG pathway analysis noted that there were a large number of down-regulated phosphorylated proteins a/ggregating in multiple cascades, including Spliceosome, RNA transport, Leishmaniasis, Ribosome biogenesis, IL−17 signaling pathway, Salmonella infection and other signaling pathways after TMP treatment. It is worth noting that in the TMP group, TMP down-regulated seven main phosphorylated proteins (fold change ≥1.5), respectively Integrin α-4(Itga4), Heat shock protein α (HSP90α), NF-kappa-B-activator(Act), TNF receptor-associated factor (TRAF), caspase-8, Transcription factor AP-1(Jun), Prostaglandin G/H synthase 2 (Ptgs2) (Figure 6C). The results of the Gene Ontology (GO) enrichment analysis (Figure 6D) showed that the down-regulated genes were gathered richly in mRNA processing, ribonucleoprotein complex biogenesis, and RNA splicing. Considering that inflammation is accompanied by the transcription and expression of a large number of inflammatory factors or related proteins, it suggests that TMP might inhibit protein transcription and translation in inflammatory responses.

HSP90α, Act, TRAF are closely related proteins involved in the NFκB signaling pathway. Specifically, the average ratio of HSP90α protein expression in the LPS and the Control group was 5.37, while the TMP group expression was 20% of that in the LPS group; after being stimulated by LPS, the TRAF protein in the cells of LPS group increased 3.34 times to that of the control group, but after TMP treatment, it decreased by 47%. More importantly, the excessive activation induced by LPS of the decisive protein Act acting as an upstream molecule of the NFκB signaling pathway were also inhibited under the treatment of TMP, and the phosphorylated Act protein could be reduced to 15% of that expressed in the LPS group. To a certain extent, this proved that TMP had an inhibitory effect on NFκB signaling pathway.

Furthermore, we explored the potential up-regulated pathway induced by TMP treatment. What surprised us was that the up-regulated phosphorylated proteins were enriched in leukocyte transendothelial migration pathway. For instance, the Claudin protein significantly increased by 15 folds after TMP treatment. Claudin acts as the most important components of the tight junctions, thus promoting the regeneration of blood vessels and the restoration of mucosal barrier and preventing the leukocyte from infiltrating into the tissue.

### TMP Inhibited Inflammation Through Inhibiting TLR4/NFκB Signaling Pathway

**FIGURE 7:**
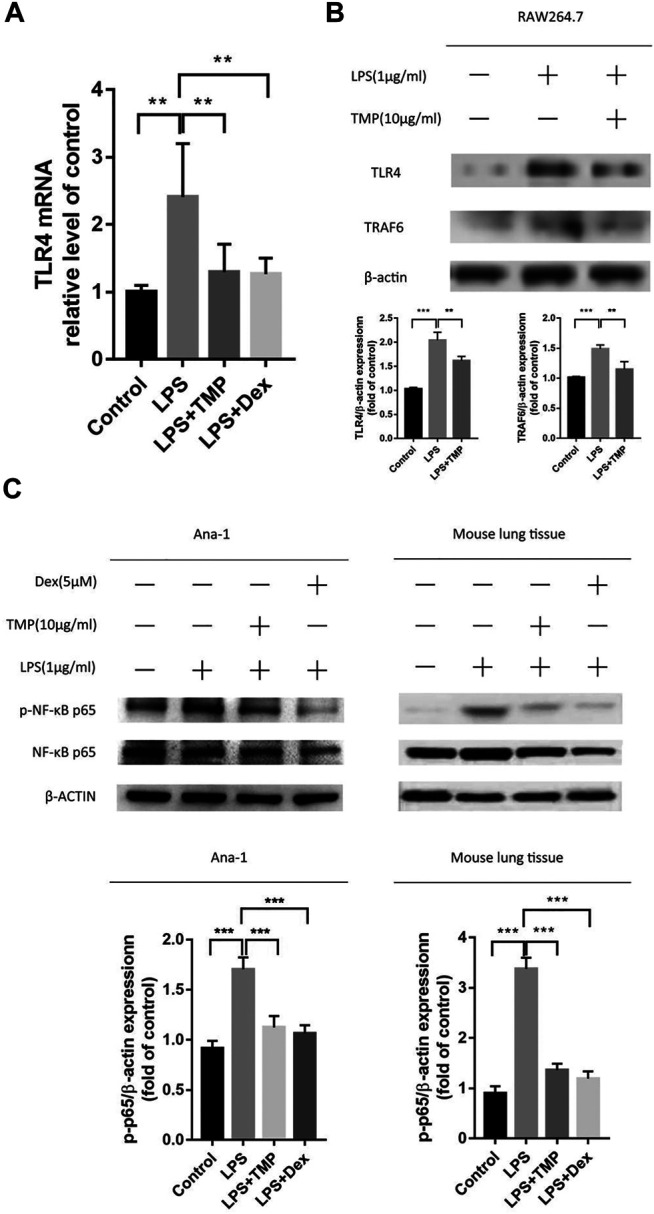
TMP inhibited inflammation through inhibiting TLR4/NFκB signaling pathway (*n* ≥ 3). C57BL/6J male mice (18–22 g) aged 6–8 weeks were randomly divided into the control group, the LPS group, the TMP group and the Dex group. **(A)** Except for the control group, mice were given an intraperitoneal injection of LPS for 6 h, and lung tissues were taken for RNA extraction. After that, the mRNA expression of TLR4 in the lungs was detected by qPCR. **(B)** RAW264.7 cells were treated in previous mentioned ways, and the protein expression of TLR4 and TRAF6 were examined. **(C)**After the above-mentioned corresponding drug treatments, lung tissue and Ana-1 cells were collected for protein extraction, then the purpose protein expression level of NFκB p-65, p-p65 were tested through Western Blot experiment. The figure below showed the quantitative data of p-65 and p-p65 protein expression. *p < 0.05, **p < 0.01, ***p < 0.001.

The results of proteomics from mass spectrometry experimental predicted that to some extent, the inhibitory effect of TMP was acted on the NFκB inflammation signaling pathway. In order to further confirm whether TMP inhibited LPS-induced inflammation and the therapy target was based on inhibiting the excessive activation of NFκB signaling pathway, we observed the expression of NFκB p-P65 protein *in vitro* and *in vivo* using qRT-PCR and Western Blot experiments. It was concluded that LPS treatment could significantly up-regulate the expression levels of TLR4, TRAF6, and p-p65 *in vivo* and *in vitro*, and TMP could significantly decrease the expression levels of TLR4, TRAF6 and p-p65 (Figures 7A–C). Therefore, combined with the above-mentioned mass spectrometry results, we concluded that TMP inhibited the expression of TLR4 and TRAF6 acting as the upstream molecules and then prevent the phosphorylation of NFκB p65 transcription factor moving into the nucleus.

### TMP Inhibited the Activation of NLRP3 Inflammasome and Caspase-1 Induced Pyroptosis

**FIGURE 8:**
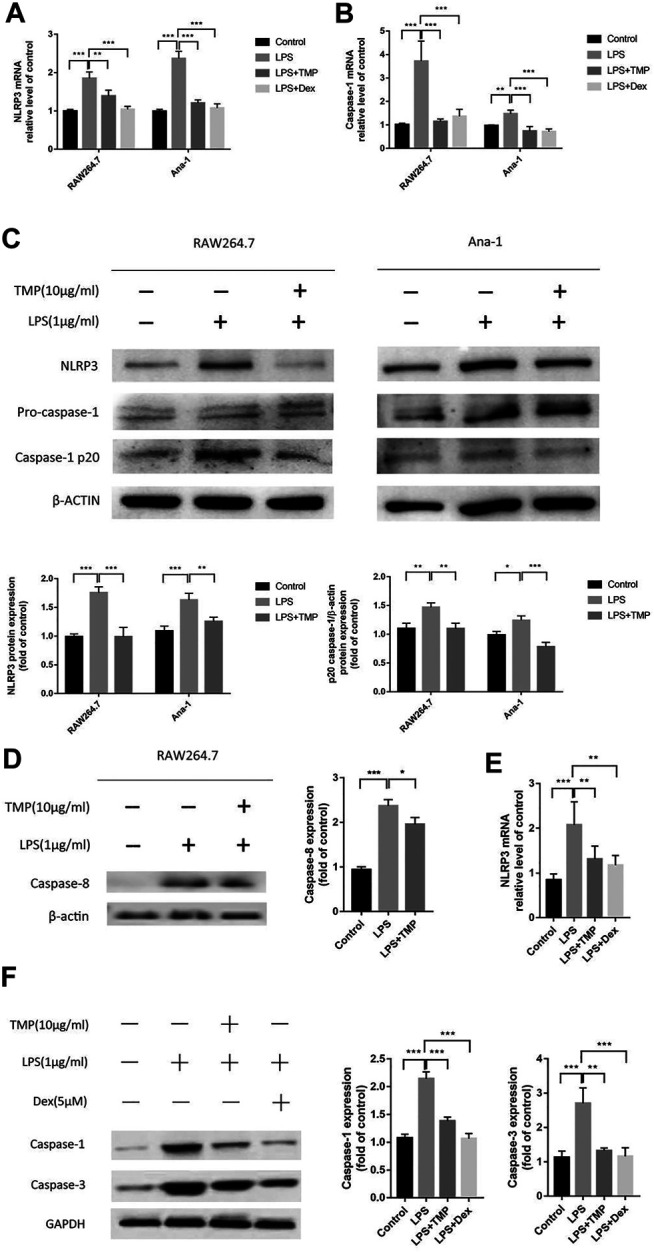
TMP inhibited the activation of NLRP3 inflammasome and caspase-1 induced pyroptosis (*n* = 3). TMP (10 μg/ml) or Dex (5 μM) and LPS (1 μg/ml) were co-treated with RAW264.7 or Ana-1 cells for 24 h **(A, B)** qRT-PCR analysis of NLRP3, caspase-1 mRNA expression. **(C,D)** After co-treatment of RAW264.7 or Ana-1 cells with LPS and TMP, Western Blot was used to detect the expression of NLRP3, caspase-8 and caspase-1/P20 protein. The figure below showed the quantitative data of NLRP3 and caspase-1/p20 protein expression. **(E)** *In vivo,* after treatment with TMP or Dex, lung tissue was taken for qRT-PCR to detect the gene expression of NLRP3. **(F)** After extracting protein from lung tissues, we performed Western Blot protein quantification experiment on caspase-1 and caspase-3. The right picture showed the quantitative data of caspase-1 and caspase-3 protein expression. Averages ± SD were shown. *p < 0.05, **p < 0.01, ***p < 0.001.

According to the above experimental results, we found that TMP could play an important role in addressing the issue of excessive releases of IL-1β and IL-18 from macrophages. Considering the close relationship between the releases of IL-1β and IL-18 and pyroptosis, we speculated that TMP may have the effect of inhibiting the pyroptosis of macrophages. In order to confirm this view, we used qRT-PCR experiments to show that TMP inhibited the activities of NLRP3 and caspase-1 (Figures 8A,B).Moreover, the results of Western blot experiments also showed that TMP could significantly inhibit the overexpression of NLPR3 and cleaved-caspase-1 induced by LPS (Figure 8C). Furthermore, the amount of P20-caspase-1 protein reflects the activation of caspase-1 precursor (Xi et al., 2016). Combining with the aforementioned experiments that TMP inhibited the release of IL-1β and IL-18, we believe that TMP has anti-pyroptosis effects in two types of macrophages (especially RAW264.7 cells). What’s more, using Western blot, we confirmed the result of protein profiling analysis that TMP reduced the expression of caspase-8, which restricted the apoptosis pathway in its downstream (Figure 8D). *In vivo* experiments, we also found that TMP can cut down the overexpression of caspase-1 and caspasae-3 induced by LPS (Figures 8E,F), which further revealed that TMP had the effects of reducing cell pyroptosis and even apoptosis.

## Discussion

TMP is an active substance extracted from chuanxiong of Umbelliferae plant. However, the therapeutic effect of TMP in ALI is still unclear. In this study we confirmed that TMP could significantly improve the survival rate of mice with LPS-induced ALI, alleviate the infiltration of inflammatory cells in lungs, and reduce the protein concentration, total cell number, neutrophils number and myeloperoxidase (MPO) activities in BALF *in vivo*. In addition, TMP inhibited pyroptosis and apoptosis of macrophages *in vivo* and *in vitro*. These results provided a reliable basis for the feasibility of TMP treatment for ALI.

It is generally accepted that macrophages are divided into two active subgroups, M1 and M2 (Nahrendorf and Swirski, 2016). M1-type macrophages are generally considered to be involved in inflammatory process, which are stimulated by LPS and/or IFNγ and produce TNFα, IL-1 and NOS relying on STAT1; M2-type macrophages are related to inflammation regression, which is stimulated by IL-4 and produce arginase and enhance Mrc and Ym1 relying on STAT6. In this study, the mRNAs of IL-6 and iNOS, flow cytometry of CD80^+^ and CD86^+^, and immunofluorescence of iNOS were used to measure the M1-type macrophages and mRNA of CD206, flow cytometry of CD206+, and immunofluorescence of CD206 were used to measure the M2-type macrophages. *In vivo,* due to the higher match of CD68 with iNOS in LPS group, we concluded that during acute lung inflammation, the M1-type macrophages would be absolutely dominant, which had a strong staining and thus led to the CD68 and iNOS duplicate positive staining. In general, according to the *vitro* and *in vivo* experiments, it was shown that TMP could inhibit M1 polarization significantly and slightly promoted M2 polarization. M2 polarization was not so significantly *in vivo*, which might be correlated with a short treatment time.

Protein profiling analysis further revealed the signal network and molecular mechanism involved in TMP. Hsp90, Act and TRAF proteins abundance were down-regulated by TMP which are all involved in the regulation of TLR-MyD88-NFκB signaling pathway. Chaperone proteins of Hsp90 family are involved in TLR signaling pathway and thus could be targeted by anti-inflammatory drugs (Wang et al., 2016). Hsp90 and Act proteins are also involved in IL-17 and MAPK signaling pathway. Hsp90 assists the folding and construction of Act protein and maintains its stability. Combined with TRAF, Act plays roles in multiple pathways, including activating TRAF6 to promote NFκB to move into nucleus (Amatya et al., 2017; McGeachy et al., 2019). JNK and AP-1 that were down-regulated by TMP were downstream components of the MAPK signaling pathway. JNK phosphorylation causes the transcription factor AP1 into nucleus, activating expression of iNOS, COX-2, PGE2 and pro-inflammatory factors TNF-α, IL- six and IL-1 (Subedi et al., 2019). Because of the focus on cell pyroptosis and apoptosis, we did not verify these signaling pathways individually, nevertheless, we provided the interesting and potential therapy target of TMP on acute inflammation for further research.

Pyroptosis is a programmed cell death with inflammation induction, which was officially named in 2001. Pyroptosis was originally thought to be a caspase-1-dependent pathway(Cookson and Brennan, 2001; Shi et al., 2015), but later studies found that caspase-4/5/11 could also activate pyroptosis (Fink and Cookson, 2005). Classical pyroptosis pathway is generally shown as an inflammasome complex containing caspase-1 precursors was firstly formed by inflammasome sensors NLRP3, AIM2 or Pyrin combining with inflammasome adaptor protein ASC, and that precursor of caspase-1 is then sheared to produce active caspase-1, leading to cleavage of the substrate Gasdermin D and production of N-terminal fragment of Gasdermin D and pyroptosis finally. The unclassical pyroptosis pathway is that caspase-4/5/11 directly lyse Gasdermin D to induce cell pyroptosis. It was reported that LPS could regulate NLRP3/ASC/caspase-1 inflammasome complex to activate lung macrophage pyroptosis through p38 MAPK pathway in LPS-induced ALI model (Kovarova et al., 2012; Wu et al., 2015; Li et al., 2018; Zeng et al., 2019). NLRP1 was also found to be involved in macrophage pyroptosis in ALI (Kovarova et al., 2012).

In this study, both the transcription and protein expression of NLRP3 and caspase-1 and active caspase-1 increased significantly after LPS treatment, which is consistent with previous studies. However, it was interesting that Ana-1 cells showed a large amount of IL-1β transcription while a relatively low release of IL-1β and that RAW264.7 cells performed less expression of IL-1β mRNA while released a large amount of IL-1β. It was found that the release of IL-1β and IL-18 and the occurrence of pyroptosis were dependent on activated caspase-1and GSDMD. Only IL-1β and IL-18 were cleaved by activated caspase-1 could be released (He et al., 2015). Such different phenomena in these two types of macrophages could be explained that pyroptosis of Ana-1 cells was dominated by non-classical pathways so that little activated caspase-1 could lead to release of IL-1β; while pyroptosis of RAW264.7 cells was dominated by classical pathway that activated caspase-1 to cut and release IL-1β and IL-18.

According to recent studies, caspase-1 caused cell apoptosis in GSDMD-deficient cells, accompanied by caspase-3 activation (Tsuchiya et al., 2019). caspase-3 cleavage triggered by AIM inflammasomes required upstream caspase-1 in caspase-8-deficient macrophages (Sagulenko et al., 2018). Therefore it was suggested that caspase-1 might be an upstream molecule involved in activating caspase-3 as a link between pyroptosis and apoptosis. This study found that the mechanisms of TMP to inhibit pyroptosis may through inhibiting TLR4/NFκB/NLRP3/caspase-1 and causing reduced releases of IL-1β and IL-18. Similarly, lower caspase-1 cleavage in Ana-1 cells than that in RAW264.7 cells also supported our hypothesis that pyroptosis of RAW264.7 cells was dominated by the classic caspase-1-dependent pathway and that of Ana-1 cells was by non-classical pathways. In addition, protein profiling analysis showed that caspase-8 protein was down-regulated by TMP. Caspase-8, as a molecular switch in programmed cell death, directly activates caspase-3 in exogenous apoptosis, or participates in endogenous apoptosis depend on mitochondrial pathway. In mitochondrial pathway, Bax/Bak induced potential changes of mitochondrial membrane, leading to mitochondrial membrane swelling and rupturing, which resulted in releasing cytochrome C and activating caspase-9 with caspase-3 activated in turn (Kandasamy et al., 2003). Expression of caspase-8 can also trigger the formation of ASC and activation of caspase-1 in case of pathogen infection (Newton et al., 2019).

In addition to the relationship between TLR4/NFκB signal pathway and apoptosis, it has been proposed that TRIF is one of the crucial protein in TLR4/NFκB signal pathway that can mediate apoptosis (Kaiser and Offermann, 2005). This apoptotic pathway has been shown to be active for TLR4, and to be responsible for bacterial-induced apoptosis of infected macrophages which involves FADD and caspase-8 acted as effector molecules (Han et al., 2004). Combined with our results that LPS induced apoptosis and TMP decreased apoptosis, it was suggested that TMP might directly inhibit cell apoptosis and pyroptosis by down-regulating TLR4/TRAF6/NFκB/NLRP3/caspase-1 and inhibiting TLR4/caspase-8/caspase-3 pathway.

Our study also found that TMP could significantly promote some proteins such as Claudin protein in leukocyte transendothelial migration pathway, which is closely correlated with the regeneration of blood vessels and the recovery of mucosal barrier. In the follow-up experiments, we will establish *in vivo* and *in vitro* models to further prove the wound repair of TMP on epithelial cells and vascular endothelial cells, so as to fully understand the clinical application of TMP in ALI.

**FIGURE 9:**
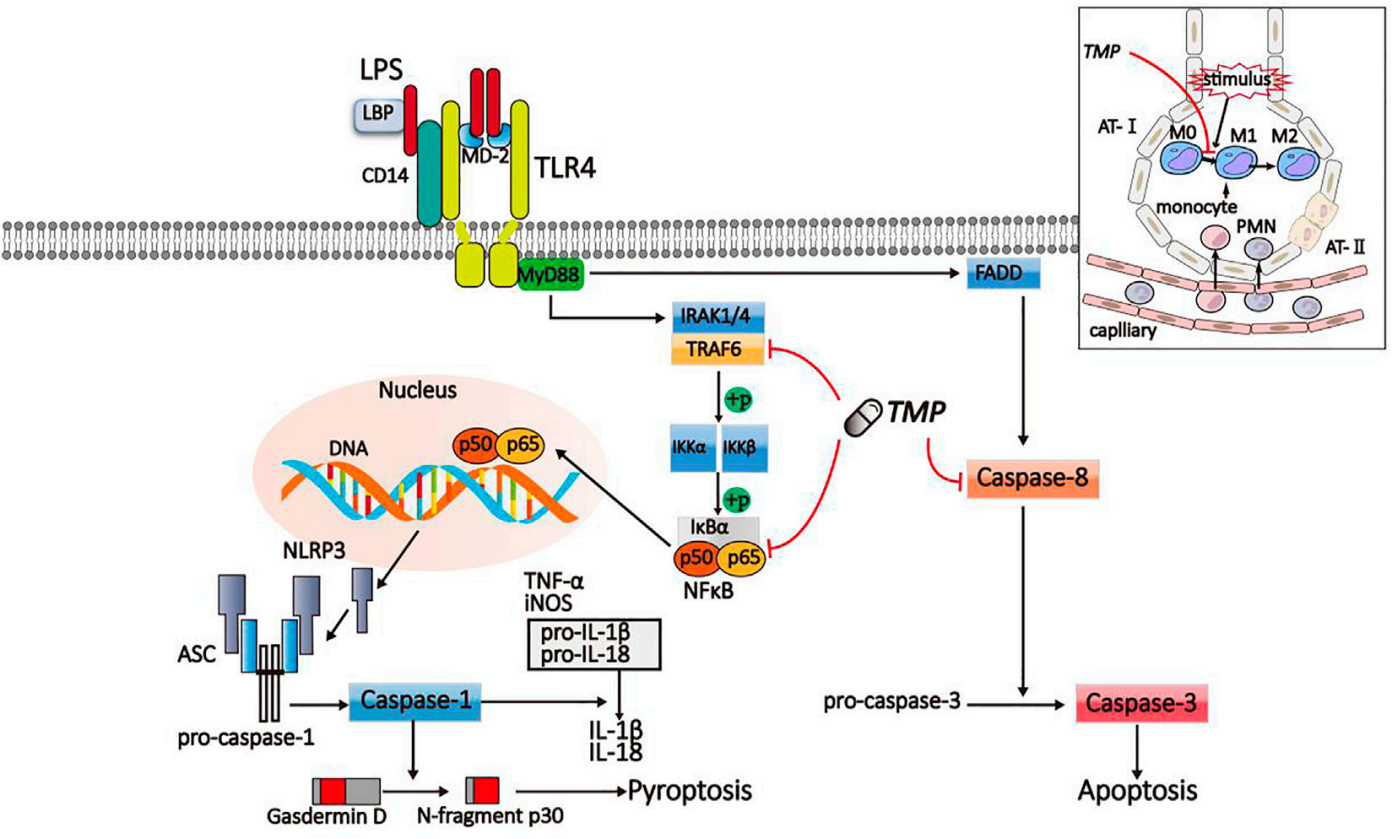
Schematic diagram of TMP on inflammatory signaling pathway. LPS activated the TLR4/TRAF6/NFκB/NLRP3/caspase-1 and TLR4/caspase-8/caspase-3 signaling pathway to induce pyroptosis and apoptosis. The signaling pathways were suppressed by TMP.

In summary, TMP could reduce pyroptosis and apoptosis of alveolar macrophage by inhibiting the TLR4/TRAF6/NFκB/NLRP3/caspase-1 and TLR4/caspase-8/caspase-3 signaling pathways, reducing the releases of pro-inflammatory factors, the polarization of M1 macrophage, and the formation of inflammasomes link to figure (Figure 9).

## Data Availability

The raw data supporting the conclusion of this article will be made available by the authors, without undue reservation.

## Abbreviation

ASC, apoptosis-associated specklike protein; AT-Ⅰ, alveolar epithelial type II cells; AT- II, alveolar epithelial type II cells; FADD, Fas-associating protein with a novel death domain; IκK, inhibitory-kappaB kinase; IκB, inhibitors of NF-kappaB; IL, interleukin; IRAK, interleukin-1 receptor-associated kinase; LBP, lipopolysaccharide binding protein; LPS, lipopolysaccharide; MD-2, myeloid differentiated protein; MyD88, myeloid differentiation primary response gene 88; NFκB, nuclear factor kappa-B; NLRP, nod-like receptor protein; PMN, polymorphonuclear neutrophil; TLR, Toll-like receptor; TMP, Tetramethylpyrazine; TNF, tumor necrosis factor; TRAF, TNF receptor associated facto
